# Antitumor Immunity Exerted by Natural Killer and Natural Killer T Cells in the Liver

**DOI:** 10.3390/jcm12030866

**Published:** 2023-01-21

**Authors:** Hiroyuki Nakashima, Manabu Kinoshita

**Affiliations:** Department of Immunology and Microbiology, National Defense Medical College, Saitama 359-8513, Japan

**Keywords:** NK cells, NKT cells, tumor immunity, innate immunity

## Abstract

The liver plays crucial roles in systemic immunity and greatly contributes to the systemic defense mechanism. Antitumor immunity in the liver is especially critical for the defense against systemic tumor cell dissemination. To achieve effective defense against metastatic tumor cells, liver immune cells with powerful cytotoxic activities construct a potent defense mechanism. In the liver, as compared with other organs, there is a significantly more intense percentage of innate immune lymphocytes, such as natural killer (NK) and NKT cells. These characteristic lymphocytes survey the portal blood transferred to the liver from the alimentary tract and eliminate malignant cells with their robust cytotoxic ability. Additionally, with their active cytokine-producing capacity, these innate lymphocytes initiate immunological sequences by adaptive immune cells. Therefore, they are crucial contributors to systemic antitumor immunity. These attractive immune cells help conduct a fundamental investigation of tumor immunity and act as a target of clinical measures for cancer therapies. This review discusses the mechanisms of these innate lymphocytes regarding recognition and cytotoxicity against tumor cells and the possibility of clinical applications for therapeutic measures.

## 1. Introduction

The liver is the largest organ in the body and is currently recognized as an immune organ due to its characteristic vascular structure and numerous innate immune cells. The portal vein collects whole venous blood drained out from the alimentary tract, and the blood is filtered through the liver before entering the systemic vascular system, which means that external pathogens and malignant cells from the gut are processed in the liver. Therefore, the liver is the front line of the body’s defense mechanism, tackling various kinds of pathogens transferred by the bloodstream; for this purpose, a liver-specific immune network is constructed by characteristic immune cells.

Antitumor immunity in the liver is an essential function that is exerted by innate immune lymphocytes, such as natural killer (NK) and NKT cells. Compared to other innate immune cells, such as Kupffer cells and neutrophils, NK and NKT cells have significant amounts of cell-killing effector materials such as perforin/granzyme. Therefore, their primary function is eliminating malignant cells and virus-infected cells. These cells are classified into large granular lymphoid cells according to their structure. In contrast to conventional lymphoid cells, they have granules in their intracellular space that offer distinct functions. In immune organs such as the spleen and lymphoid nodule, the number of NK and NKT cells represents a small and relatively rare cell population. In the liver, however, these two cell types comprise almost one-third of the total lymphoid cells and are a major cellular component of the immune system. Their functions have been discussed for decades, and their major function is antitumor or anti-viral immunity. NK and NKT cells can recognize damaged or abnormal hepatic cells or cancer cells transferred from the alimentary tract and eliminate them from the parenchymal space, which contributes to antitumor and anti-viral immunity.

The mechanisms of effector functions against tumor cells go through several steps, such as the recognition and cytotoxicity of target tumor cells. This review focuses on the mechanisms of tumor cell elimination exerted by innate immune cells in the liver and possible therapeutic applications to human cancer immunotherapy.

## 2. NK Cells

### 2.1. Overview

In 1975, NK cells were uncovered as lymphoid cells with natural cytotoxicity against tumor cell lines [1]. They exist in various lymphoid and non-lymphoid organs, and the lung and liver are the most abundant organs, as NK cells comprise around 6% of mononuclear cells in the liver and 10% in the lung [2]. Given the high chance of metastasis from various cancer cells, an abundant number of these effector cells reflect that the lung and the liver possess significant roles in the systemic defense against tumor cells. NK cells are the most potent antitumor immune effector and have a considerable amount of perforin/granzyme for the induction of apoptosis against target cells. Not only do they direct effector function, but they can also indirectly contribute to antitumor immune reactions against tumor cells. NK cells are activated with various pathogen-associated molecular patterns (PAMPs) and secrete cytokines such as IFN-γ to enhance various kinds of immune reactions. The human counterpart of this cell population is CD56-positive and CD3-negative cells, which comprise about 30% of the human liver [3].

### 2.2. Recognition of Tumor Cells

As NK cells do not express antigen-specific receptors with a diverse repertoire that can detect various kinds of individual proteins; they do not recognize specific tumor cells. Instead, NK cells distinguish tumor cells with receptors that can detect irregular signals expressed on the target cells, and the receptors are classified into activating and suppressing functions.

**Figure 1:**
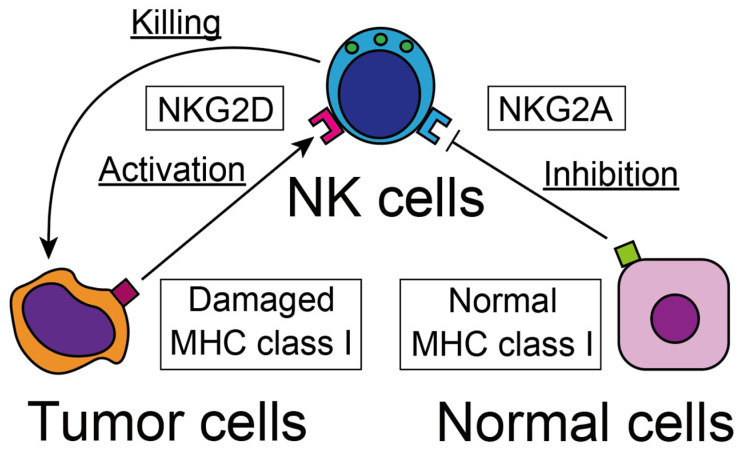
NK cells recognize tumor cells with their activating and inhibitory receptors. Tumor cells that have lost standard MHC class I are the best targets for NK cells.

Among the activating receptors are the Fc receptors CD16 and CD62, which recognize specific antibodies attached to tumor cells. Through these Fc receptors, NK cells can exert efficient cytotoxic activity against antibody-coated tumor cells, and this process is called antibody-dependent cell-mediated cytotoxicity (ADCC) [4]. The other well-known activating receptor is natural killer group 2D (NKG2D), which can detect damaged and stressed cells (Figure 1) [5]. The receptor detects various tumor cell-related antigens, most of which are a degraded form of major histocompatibility complex (MHC) class I antigens, such as MHC class I chain-related protein A and B (MICA and MICB), UL16-binding proteins in humans, retinoic acid early inducible-1 (RAE1) family protein, and mouse UL-16-binding protein-like transcript 1 (MULT-1) in mice [5]. Moreover, NKG2D expression is up-regulated by various kinds of cellular stresses [6], such as heat shock [7] and DNA damage-related antigens [8]. Through the NKG2D receptor, NK cells detect and eliminate degraded cells under these cellular stresses.

The normal MHC class I antigen, which is expressed on intact cells, inhibit effector function on NK cells, therefore, tumor cells lacking MHC class I are vigorously eliminated by NK cells [9]. Because NK cells exert magnificent activity to eliminate tumor cells without self-recognition molecules such as MHC class I, this mechanism is called the “missing self” theory [10]. Natural killer group 2A (NKG2A) is the main receptor for detecting the intact MHC class I antigen and the most effective receptor signal that inhibits cytotoxicity against intact cells [11]. Therefore, infected, degraded cells and tumor cells that have lost normal MHC class I are the best targets for NK cells and are eliminated from the tissue. Additionally, excessive activation of NK cells can exert a potential cytotoxic effect, even on normal cells with intact MHC class I. Therefore, the balance between activating and inhibitory receptors is the key fate-determination factor of NK cells.

### 2.3. Cytotoxicity against Tumor Cells

Immune cells with potential cytotoxic activity use several kinds of effector molecules to induce apoptosis on target cells. One of the most effective mechanisms is the perforin-granzyme pathway, in which effector lymphocytes form pores on a cellular surface with “perforin” and transfer serin protease “granzyme” to induce an apoptosis reaction against target cells [12]. Ohkawa et al. compare the amount of intracellular perforin-granzyme content with the cytotoxic activities of human counterpart cells of mouse NK cells and CD8-positive conventional T cells [13]. According to their results, CD56-positive NK cells marked the highest perforin content and cytotoxic activity against MHC class I negative tumor cells. These results suggest that the primary pathway of cytotoxic potential for NK cells is via perforin-granzyme.

Recent research, however, has revealed a brand-new perforin-related cytotoxic mechanism. Among the granzyme protein family subclasses, granzyme B is the primary molecule for the induction of apoptosis, and the other protein, “granzyme A”, has different cytotoxic activity. Granzyme A can induce pyroptotic death against tumor cells with gasdermin B protein, which is expressed abundantly in epithelial cells in the gastrointestinal tract [14]. Pyroptosis is an entirely different mechanism of cell death, distinct from apoptosis in the point of inducing inflammatory reactions to facilitate immune responses [15]. Gasdermin B protein is abundantly expressed in epithelial cells of the alimentary tract and highly susceptible to pyroptosis induced by granzyme A. This mechanism is essential for the elimination of gastrointestinal tumor cells. The other cytotoxic effector molecules on NK cells are the TNF receptor family, including Fas/FasL and TNF-related apoptosis-inducing ligand (TRAIL)/DR4 and DR5 interaction [16]. Additionally, NK cells can cause tumor cell death by a direct effect of cytokines such as IFN-γ and TNF-α [17].

### 2.4. Possible Therapeutic Strategy against Human Cancer

The therapeutic applications of NK cells against cancer patients are divided into two strategies: one is to activate NK cells in the patients to recruit them into cancer focus and exert cytotoxicity against malignant cells, and the other is isolating NK cells from the patients and transferring them back after adequate stimulation in vitro. In line with this strategy, IL-2 cytokine therapy for activating intrinsic NK cells, combined with injection of ex vivo stimulated NK cells, was applied to clinical studies [18]. However, several severe side effects were reported, and the clinical application was abandoned [19]. The systemic administration of cytokine can up-regulate the functions of immune cells; however, it also induces a cytokine storm and risks the lives of patients, namely, the artificial activation of the innate immune system can be a double-edged sword. The IL-2 receptor has three subclasses, α, β, γ, and the effect induced by intracellular signals for each receptor subclass are totally different. The IL-2Rα receptor (CD25) is expressed on CD4-positive T cells and induces expansion of regulatory T cells; in contrast, the IL-2Rβ receptor (CD122) is expressed on NK cells and is involved in NK cell activation. For effective NK cell-targeted cytokine therapy, selective activation of IL-2Rβ is preferable, and several effective therapeutic measures have been developed. The “IL-2 superkine” with 200× affinity against IL-2Rβ is one example, as this agent can effectively activate NK cells [20] and reverse the desensitization of NK cells against tumor cells [21].

The antitumor activity of NK cells is regulated by balancing between stimulating and inhibiting receptors. The appropriate modulation of this balance can then efficiently up-regulate the antitumor defense. The molecular targeting therapy aiming to block the inhibitory NK receptor can up-regulate the antitumor effector function by NK cells. For example, “Monalizumab”, the monoclonal antibody targeting NKG2A, can augment NK cell cytotoxicity and improve the survival rate of the experimental mouse model [22]. This promising report suggests the future use of immunotherapy induced by NK cells, which have significant cytotoxicity potential against tumor cells. However, as with classical cytokine therapy, careful investigation is needed for clinical application to avoid severe side effects. According to the clinicaltrials.gov (National Library of Medicine, Bethesda, MD, USA), more than seventy clinical trials of NK cell-related immunotherapies are underway worldwide (Supplementary Materials).

NK cells are directly activated by pathogen-associated molecular patterns (PAMPs) receptors, and PAMPs receptor agonists can activate NK cells and augment the antitumor cytotoxic effect. The toll-like receptor (TLR) 7/TLR8 agonist “Imiquimod” can exert an antitumor effect on MHC class I deficient tumor cells with the assistance of CD4 + T cells [23]. This agent has already been applied to clinical use for the treatment of papillomavirus-related verts [24].

## 3. NKT Cells

### 3.1. Overview

The elucidation of unusual T lymphocytes with invariant T cell receptors was a significant focus of immunology research from 1990 to 2000 [25]. For the first time, NK1 + T cells were revealed in the bone marrow [26] and seen as comprising up to 39% of bone marrow T cells [27]. At around the same time, Ohteki et al. reported that unusual T cells with intermediate T cell receptor intensity in MRL-lpr/lpr mice vigorously proliferate in the liver [28]. Moreover, there exist counterpart T cells with a skewed repertoire of T cell receptor (TCR) with Vβ8 in the normal liver [29]. The proliferation of unusual T cells with Vβ8 TCR caused the researchers to think that these T cells were extra-thymic differentiation. However, the same counterpart with the NK1 antigen was also found in the thymus from different groups, and NK1 + T cells were accepted as a thymic origin [30,31]. NK1 + T cells attracted many immunologists given their vigorous potential to secrete various kinds of cytokines, and researchers eagerly performed repertoire analyses for TCR. Most of the NK1 + T cells possess skewed TCR repertoire Vα14 Jα18 paired with Vβ7 or Vβ8.2 [32,33,34], and several groups defined this distinct T cell population in the liver and other organs as “NKT cells” [35,36]. Thereafter, the cells express the Vα14-Vβ8-Jα11 repertoire and are called type I NKT cells, while others are called type 2 NKT cells [25]. The human counterpart of this cell population is Vα24Jα18 paired with Vβ11 [32]. However, the number of T cells expressing this receptor is limited, as these cells comprise around 1% of CD56-positive T cells. Nevertheless, there are abundant CD56-positive T cells in the liver, almost the same amount as in mice [3]. Therefore, CD56-positive T cells are more consistent as NKT cells in mice. Indeed, the T cell receptor intensity on CD56-positive T cells is less than for conventional T cells, which is identical to NKT cells in mice [13].

### 3.2. Recognition of Tumor Cells

**Figure 2:**
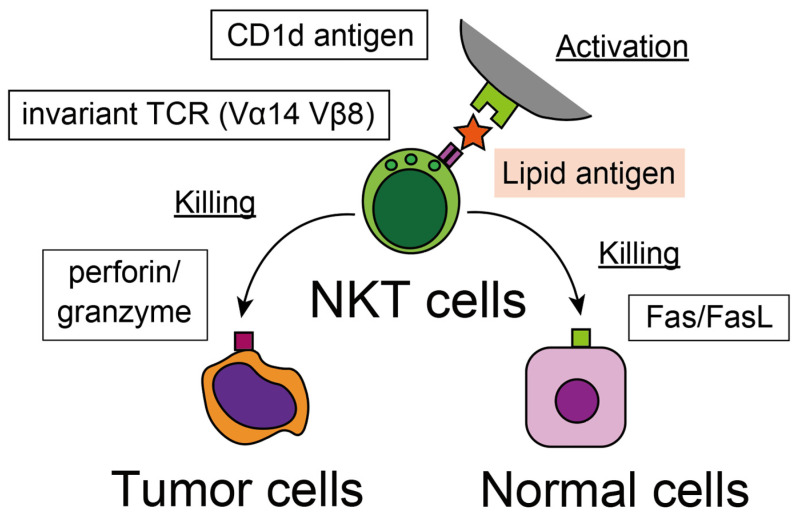
NKT cells recognize lipid-related antigens expressed on CD1d protein.

For conventional T cells, the function of the T cell receptor is to detect a wide variety of specific antigens; consequently, a significant diversity of the receptor repertoire is assembled. However, the invariant T cell receptor of NKT cells suggests that they use TCR in a different manner, possibly for detecting a wide range of antigens, such as pattern recognition receptors for PAMPs and damage-associated molecular pattern molecules (DAMPs). One of the characteristic features of NKT cells is their strict dependence on the cluster of differentiation 1d (CD1d) molecule (Figure 2) [37]. CD1d is an MHC class I-like protein expressed by different kinds of systemic cells and presents various lipid antigens to T cells, such as mycolate from mycobacteria [38]. NKT cells recognize abnormal cells, such as infected, damaged, aged, and cancerous cells expressing lipid-CD1d molecule combinations. Consistently, the metastatic potential of the mice model of breast cancer is inversely correlated with the expression of CD1d on tumor cells and the effectiveness of NKT cell recognition [39]. In a human study, the prognosis of human non-small cell lung cancer (NSCLC), correlated with the expression of CD1d on lung tissue and the induction of CD1d on the NSCLC cell line in vitro, up-regulated the iNKT cell-mediated tumor cell elimination [40]. The CD1d molecule is expressed on various kinds of cells and contributes to immunosurveillance by iNKT cells. In addition to the direct recognition of tumor cells, iNKT cells recognize lipid-CD1d combinations expressed on antigen-presenting cells (APCs), such as dendritic cells (DC) and B cells, and initiate sequential immune reaction for the more potent elimination of pathogenic cells. Interestingly, the direction of the immune response depends on the type of APC; activation by CD1d on DC induces a Th1-like reaction [41], while a Th2-like response is induced by B cells [42].

### 3.3. Cytotoxicity against Tumor Cells

Among the lipid-related ligands that attach this invariant TCR, α-galactosylceramide (α-GalCer) is the most potent NKT cell activator [43]. The administration of α-GalCer induced a potent antitumor effect that could completely block liver metastasis after tumor cell injection in mice, and this effect occurs in a Vα14-CD1d-dependent manner [44]. According to these mouse experiments, human NKT cells also exert cytotoxicity against CD1d molecule-bearing tumor cells via perforin/granzyme [45]. These attractive reports have highlighted NKT cells and influenced many immunology researchers, and precise mechanisms of antitumor immunity have been investigated. There are two major pathways for killing tumor cells by cytotoxic lymphocytes: perforin/granzyme and Fas/FasL. These two pathways were properly used according to the target cells [46]. For the elimination of tumor cells, the contribution of perforin/granzyme is more significant than Fas/FasL [46]. Similarly, α-GalCer-activated NKT cells reportedly exert their cytotoxic activity via perforin/granzyme [44,45]. However, another group has reported that the intracellular perforin content in NKT cells was lower than in NK cells, and the cytotoxic effect of NKT cells was weaker than NK cells, according to the investigation of human counterpart cells [13]. The antitumor effect of α-GalCer stimulation is exerted not only through direct cytotoxicity by NKT cells but also by the activation of NK cells, which can eliminate CD1d negative tumor cells [47]. Nakagawa et al. report that the abrogation of NK cells by AsialoGM1 antibody abolished improvement in both in vitro cytotoxicity and in vivo survival of metastatic tumor models of NK-sensitive and resistant tumors [48]. This report indicates that the indirect antitumor effect evoked by activated NK cells is more significant than direct cytotoxicity by activated NKT cells. However, activated NKT cells by α-GalCer induced apoptosis of hepatocytes in a TNF-Fas-FasL-dependent manner, especially in aged mice [49]. Interestingly, this effect is independent of antitumor immunity induced by the IFN-γ-perforin-granzyme pathway; because cytotoxicity against liver and tumor cells is independently regulated, hepatotoxicity is blocked by prior administration of anti-TNF or anti-FasL antibodies without attenuating cytotoxicity against tumor cells [50]. After NK cell activation, NKT cell activation by α-GalCer then up-regulates the cytotoxicity of CD8 + CD122 + T cells, and it can induce memory T cells for the effective elimination of tumor cells [51]. As a result, α-GalCer induces not only the upregulation of the direct cytotoxicity effect of NKT cells but also robust and durable antitumor immunity sequences that can cover various kinds of tumor cells. Overall, NKT cells are the crucial conductor to orchestrate antitumor immunity.

### 3.4. Possible Therapeutic Strategy against Human Cancer

Unlike in mice, the number of human iNKT cells with Vα24 Vβ8.2 TCR is much smaller than the other classical T cells. Despite their limited cell number, considerable evidence from different groups suggests the enormous contributions of NKT cells to antitumor immunity. The number and function of iNKT cells strictly correlates with the prognosis of various human cancer patients [52], including prostate [53], gastrointestinal [54], and hematopoietic malignancies [55]. This evidence has attracted researchers and encouraged them to investigate the therapeutic potential of iNKT cells for human cancer.

The activation of iNKT cells by α-GalCer exerts a potent antitumor effect upon metastatic tumor models in mice and is expected to be an efficient antitumor immune therapy [44]. It is noteworthy that α-GalCer injections exert a significant antitumor effect, even after several days of tumor cell inoculations, which strongly suggests their potential for curative clinical usage [56]. However, clinical trial studies on human solid cancer patients with α-GalCer have not shown promising results [57]. In their study, Giaccone et al. emphasize that the number of iNKT cells in peripheral blood decreased in tumor-bearing patients, and there was a strict inverse correlation between the number and the therapy effectiveness. The limited number of iNKT cells is the critical deterrent in this strategy, and various modifications of α-GalCer therapy have been proposed so far. The adoptive transfer of α-GalCer-loaded DC can improve the prognosis of myeloma [58] and neck cancer [59]. However, previous researchers have not been able to attain sufficient remission effects that are suitable for clinical application. Interestingly, recent studies have reported promising curative results using chimeric antigen receptor (CAR) T cell technology. CAR T cells targeting CD19 antigen can provide a curative outcome in refractory B cell lymphoma [60]. This technology was converted to the iNKT cells and created “CAR-NKT cells”, which concomitantly express CAR and invariant T-cell receptors. Human invariant NKT cells were isolated from peripheral blood with a magnetic sorting technique. The obtained cells were transduced with CAR lentivirus, activated in vitro for seven days, and adoptively transferred to the host. The adoptive transfer of CAR-NKT cells can induce a favorable antitumor effect on refractory B cell lymphoma without causing graft versus host reaction [61]. This antitumor effect is augmented by α-GalCer treatment and provides a stronger therapeutic effect than the classical CAR-T cell therapy [62]. Similarly, CAR-NKT cell therapy targeting GD2 antigen expressed by melanoma and neuroblastoma can provide promising results and is expected to produce successful clinical applications [63]. Clinical trial studies of CAR-NKT cell therapy against B cell lymphoma and neuroblastoma are underway in the United States. (Table 1). Although this therapy is expected to be an effective immunotherapy using NKT cells, the problem is the limited number of iNKT cells in peripheral blood cells. To obtain a sufficient number of NKT cells, a large amount of blood is necessary for the curative measure. The ex vivo expansion technique will be the key to overcoming this limitation.

While the limited cell number has hampered the application of iNKT cells in human clinical procedures, there are abundant T cells co-expressing NK cell-related antigen CD56 in the liver. Moreover, their cell number is closely correlated with the fibrosis stage and hepatocarcinogenesis of chronic hepatitis C [3]. This report strongly suggests that non-invariant, type II NKT cells abundantly exist in humans and exert a significant role in antitumor immunity. The perforin content of CD56-positive T cells is higher than conventional T cells, suggesting their substantial effector activity [13]. Research work aimed at the clinical application of NKT cells has focused on invariant type I NKT cells. However, the composition of T cells with NK-related antigen in humans is rather complex compared to those in mice. Therefore, the elucidation of type II NKT cell function is quite important, and they can be another strategy for effectively applying NKT cells to antitumor immune therapy.

In the current medical procedure, immune checkpoint inhibitors such as programmed cell death (PD)-1 antibodies demonstrate favorable clinical results. However, these checkpoint inhibitors have not provided significant remission effects in primary liver cancer, such as hepatocellular carcinoma and cholangiocarcinoma, suggesting that currently available inhibitors do not directly affect NK and NKT cell functions [64]. However, combination therapy with NK/NKT cell adoptive transfer therapy may augment their effect on each other and increase the curative effects. Further investigations will be expected.

## 4. Concluding Remarks

NK and NKT cells are non-antigen specific innate lymphocytes that enormously contribute to antitumor immunity. They have significant surveillance functions and produce cytotoxic activity against tumor cells in various non-lymphoid organs. They particularly accumulate in crucial filtering organs such as the liver and lung and exert their functions to protect against systemic tumor cell dissemination in cancer patients. Considering their potential cytotoxic activity, NK and NKT cells are promising candidates for cancer immunotherapy. Understanding their physiological function can elucidate the mechanisms of antitumor immunity and contribute to the development of efficient cancer therapy strategies. Therefore, NK and NKT cells in the liver and the lung are attractive research targets to overcome the current refractory cancer status.

## Figures and Tables

**Table 1:** Ongoing clinical trials of NKT cell-related immune therapy (clinicaltrials.gov).

**Identifier**	**Title**	**Conditions**	**Locations**
NCT05487651	Allogeneic NK T-Cells Expressing CD19 Specific CAR in B-Cell Malignancies (ANCHOR2)	NHL, Relapsed, Adult B-cell Lymphoma B-cell Leukemia	University of California, San Francisco, San Francisco, CA, USA
NCT03774654	CD19.CAR Allogeneic NKT for Patients with Relapsed or Refractory B-Cell Malignancies (ANCHOR)	Refractory B-Cell Non-Hodgkin Lymphoma	Houston Methodist Hospital, Houston, TX, USA
NCT03294954	GD2 Specific CAR and Interleukin-15 Expressing Autologous NKT Cells to Treat Children with Neuroblastoma (GINAKIT2)	Neuroblastoma	Texas Children’s Hospital, Houston, TX, USA
NCT03093688	Clinical Safety and Efficacy Study of Infusion of iNKT Cells and CD8 + T Cells in Patients with Advanced Solid Tumor	Non-small Cell Lung Cancer Small Cell Lung Cancer Pancreas Cancer	Shanghai Public Health Clinical Center, Shanghai, China
NCT02562963	Clinical Efficacy and Safety of NKT Cell Infusion in Patients with Advanced Solid Tumor	Non-small Cell Lung Cancer Gastric Cancer	Hua Xin Hospital First Hospital of Tsinghua University, Beijing, China

## Data Availability

The data regarding ongoing clinical trials were obtained from https://clinicaltrials.gov/, accessed on 21 November 2022.

## Supplementary Materials

The following supporting information can be downloaded at: https://www.mdpi.com/article/10.3390/jcm12030866/s1, File S1: ClinicalTrials.gov Search Results.
