# Supplementary material for: Antitumor Immunity Exerted by Natural Killer and Natural Killer T Cells in the Liver
Source: J Clin Med. 2023 Jan 21;12(3):866. doi: 10.3390/jcm12030866 (PMC9917438; doi:10.3390/jcm12030866)
Supplement: Supplementary file 1 [file jcm-12-00866-s001.zip › jcm-2081890-supplementary.pdf]

File S1. ClinicalTrials.gov Search Results 01/09/2023

	Title	Status	Study Results	Conditions	Interventions	Locations
1	Natural Killer (NK) Cell Therapy Targeting CD33 in Acute Myeloid Leukemia	Recruiting	No Results Available	•AML, Adult	•Drug: QN-023a<br>•Drug: Cyclophosphamid<br>•Drug: Fludarabine<br>•Drug: Cytarabine<br>•Drug: VP-16	•The first affiliated hospital of medical college of zhejiang university, Hangzhou, Zhejiang, China
2	A Study of Universal CD19-Targeted UCAR-NK Cells Combined With HSCT for B Cell Hematologic Malignancies	Recruiting	No Results Available	•B-Cell Lymphoblastic Leukemia/Lymphoma	•Biological: Anti-CD19 UCAR-NK cells	•920th Hospital of Joint Logistics Support Force of People's Liberation Army of China, Kunming, Yunnan, China
3	Clinical Study of the Safety and Efficacy of BCMA CAR-NK	Recruiting	No Results Available	•Immunotherapy<br>•Multiple Myeloma	•Drug: Chimeric Antigen Receptor NK Cell Injection Targeting BCMA (BCMA CAR-NK)	•Henan Cancer Hospital, Zhengzhou, Henan, China
4	CAR-NK Targeted CD19 for r/r B-cell Malignancies	Recruiting	No Results Available	•Adult Relapsed/Refractory B-cell Hematologic Malignancies	•Biological: CD19-CAR-NK	•The Fifth Medical Center of Chinese People's Liberation Army (PLA) General Hospital, Beijing, Beijing, China
5	A Multi-Institution Study of TGF# Imprinted, Ex Vivo Expanded Universal Donor NK Cell Infusions as Adoptive Immunotherapy in Combination With Gemcitabine and Docetaxel in Patients With Relapsed or Refractory Pediatric Bone and Soft Tissue	Recruiting	No Results Available	•Pediatric Sarcoma, Refractory<br>•Pediatric Sarcoma, Relapsed	•Biological: GEM/DOX + TGFBi expanded NK cells	•National Pediatric Cancer Foundation, Tampa, Florida, United States<br>•Nationwide Children's Hospital, Columbus, Ohio, United States
6	Natural Killer(NK) Cell Therapy for Acute Myeloid Leukemia	Recruiting	No Results Available	•AML, Adult	•Drug: QN-023a<br>•Drug: Cyclophosphamid<br>•Drug: Fludarabine<br>•Drug: Cytarabine	•Institute of Hematology & Blood Diseases Hospital, Tianjin, Tianjin, China
7	Natural Killer Cell Therapy (UD TGFbetai NK Cells) and Temozolomide for the Treatment of Stage IV Melanoma Metastatic to the Brain	Recruiting	No Results Available	•Clinical Stage IV Cutaneous Melanoma AJCC v8<br>•Metastatic Malignant Neoplasm in the Brain<br>•Metastatic Melanoma<br>•Pathologic Stage IV Cutaneous Melanoma AJCC v8	•Biological: Natural Killer Cell Therapy<br>•Drug: Temozolomide	•Ohio State University Comprehensive Cancer Center, Columbus, Ohio, United States
8	Cytokine-Induced Memory-Like Natural Killer Cells (CIML-NK) for Relapsed & Refractory Acute Myeloid Leukemia (AML)	Recruiting	No Results Available	•Acute Myeloid Leukemia	•Drug: CIML-NK Cells	•Cincinnati Children's Hospital Medical Center, Cincinnati, Ohio, United States
9	Allogenic CD123-CAR-NK Cells in the Treatment of Refractory/Relapsed Acute Myeloid Leukemia	Recruiting	No Results Available	•Acute Myeloid Leukemia Refractory<br>•Acute Myeloid Leukemia Recurrent	•Biological: CD123-CAR-NK cells	•The Fifth Medical Center of Chinese People's Liberation Army (PLA) General Hospital, Beijing, Beijing, China
10	NKG2D-CAR-NK92 Cells Immunotherapy for Solid Tumors	Recruiting	No Results Available	•Relapsed/Refractory Solid Tumors	•Biological: NKG2D-CAR-NK92 cells	•The first Affiliated Hospital of Xinxiang Medical University, Xinxiang, Henan, China
11	Study of DLL3-CAR-NK Cells in the Treatment of Extensive Stage Small Cell Lung Cancer	Recruiting	No Results Available	•SCLC, Extensive Stage	•Biological: DLL3-CAR-NK cells	•Tianjin Medical University Cancer Institute and Hospital, Tianjin, Tianjin, China
12	Safety and Efficacy of Expanded, Universal Donor Natural Killer Cells for Relapsed/Refractory AML	Recruiting	No Results Available	•Acute Myeloid Leukemia	•Biological: Universal Donor Natural Killer Cells	•Nationwide Children's Hospital, Columbus, Ohio, United States
13	Clinical Study of Cord Blood-derived CAR-NK Cells Targeting CD19 in the Treatment of Refractory/Relapsed B-cell NHL	Recruiting	No Results Available	•B-cell Non Hodgkin Lymphoma	•Biological: anti-CD19 CAR-NK	•2nd Affiliated Hospital, School of Medicine, Zhejiang University, Hanzhou, Zhejiang, China
14	CLDN6-CAR-NK Cell Therapy for Advanced Solid Tumors	Recruiting	No Results Available	•Stage IV Ovarian Cancer<br>•Testis Cancer, Refractory<br>•Endometrial Cancer Recurrent<br>•CAR NK	•Biological: Claudin6 targeting CAR-NK cells	•The Second Affiliated Hospital of Guangzhou Medical University, Guangzhou, Guangdong, China

	Title	Status	Study Results	Conditions	Interventions	Locations
15	Anti-CD19 CAR-Engineered NK Cells in the Treatment of Relapsed/Refractory B-cell Malignancies	Recruiting	No Results Available	•Acute Lymphocytic Leukemia<br>•Chronic Lymphocytic Leukemia<br>•Non Hodgkin Lymphoma	•Biological: CAR-NK-CD19 Cells	•Beijing Boren Hospital, Beijing, Beijing, China
16	Natural Killer (NK) Cells in Combination With Interleukin-2 (IL-2) and Transforming Growth Factor Beta (TGFbeta) Receptor I Inhibitor Vactosertib in Cancer	Recruiting	No Results Available	•Colorectal Cancer<br>•Hematologic Malignancy<br>•Rectum Cancer<br>•Acute Myeloid Leukemia<br>•Myelodysplastic Syndromes<br>•Acute Lymphoblastic Leukemia<br>•Chronic Myeloid Leukemia<br>•Chronic Lymphocytic Leukemia<br>•Hodgkin Lymphoma<br>•Non Hodgkin Lymphoma<br>•Myeloproliferative Syndrome<br>•Plasma Cell Myeloma	•Drug: Vactosertib<br>•Drug: Fludarabine Phosphate<br>•Drug: Cyclophosphamide<br>•Drug: IL-2<br>•Drug: Natural Killer Cells	•University Hospitals Cleveland Medical Center, Case Comprehensive Cancer Center, Cleveland, Ohio, United States
17	A Phase I, Autologous ex Vivo Expanded and Activated NK Cell, Magicell-NK, Infusion for Colon Cancer Post Resection Study	Recruiting	No Results Available	•Colon Cancer Stage I	•Biological: Magicell-NK contains NK cells suspended in 100 mL normal saline	•Chang Gung Memorial Hospital, Linkou, Taoyuan, Taiwan
18	Natural Killer (NK) Cell Therapy for B-Cell Malignancies	Recruiting	No Results Available	•B-cell Lymphoma<br>•B-cell Acute Lymphoblastic Leukemia	•Drug: QN-019a<br>•Drug: Rituximab<br>•Drug: Cyclophosphamid<br>•Drug: Fludarabine<br>•Drug: VP-16	•The First Affiliated Hospital of Medical College of Zhejiang University, Hangzhou, Zhejiang, China
19	Expanded Haploidentical Natural Killer Cells as Consolidation Strategy for Children/Young Adults With AML	Recruiting	No Results Available	•Acute Myeloid Leukemia	•Biological: Expanded haploidentical NK cells	•Belarussian Research Center for Pediatric Oncology, Hematology and Immunology, Minsk, Minsk Region, Belarus
20	Genetically Engineered Natural Killer (NK) Cells With or Without Atezolizumab for the Treatment of Non-small Cell Lung Cancer Previously Treated With PD-1 and/or PD-L1 Immune Checkpoint Inhibitors	Recruiting	No Results Available	•Advanced Lung Non-Small Cell Carcinoma<br>•Metastatic Lung Non-Small Cell Carcinoma<br>•Recurrent Lung Non-Small Cell Carcinoma<br>•Refractory Lung Non-Small Cell Carcinoma<br>•Stage III Lung Cancer AJCC v8<br>•Stage IIIA Lung Cancer AJCC v8<br>•Stage IIIB Lung Cancer AJCC v8<br>•Stage IIIC Lung Cancer AJCC v8<br>•Stage IV Lung Cancer AJCC v8<br>•Stage IVA Lung Cancer AJCC v8<br>•Stage IVB Lung Cancer AJCC v8	•Biological: Antineoplastic Immune Cell<br>•Biological: Atezolizumab<br>•Procedure: Biospecimen Collection<br>•Drug: Cyclophosphamide<br>•Drug: Fludarabine	•City of Hope Comprehensive Cancer Center, Duarte, California, United States
21	Donor Immune Cell Therapy for Acute Myeloid Leukemia	Recruiting	No Results Available	•Acute Myeloid Leukemia	•Biological: infusion of natural killer cells	•First Affiliated Hospital of Xian Jiaotong University, Xi'an, Shaanxi, China

	Title	Status	Study Results	Conditions	Interventions	Locations
22	"Phase I / II Study on Infusion of Natural Killer Cells After Haploidentical Transplantation in Pediatric Patients"	Recruiting	No Results Available	•High-risk Leukemias	•Biological: NK cells stimulated ex vivo with IL-15<br>•Biological: Alloreactive NK cells	•Hospital Clínico Universitario de Santiago, Santiago De Compostela, A Coruña, Spain<br>•Hospital Universitario Central de Asturias, Oviedo, Asturias, Spain<br>•Hospital de la Santa Creu i Sant Pau, Barcelona, Spain<br>•Hospital General Universitario Gregorio Marañón, Madrid, Spain<br>•Hospital Infantil Universitario Niño Jeús, Madrid, Spain<br>•Hospital Universitario La Paz, Madrid, Spain<br>•Hospital Clínico Universitario Virgen de la Arrixaca, Murcia, Spain<br>•Hospital Regional Universitario de Málaga (Carlos de Haya), Málaga, Spain<br>•Hospital Universitario Virgen del Rocío, Sevilla, Spain<br>•Hospital Universitario La Fe, Valencia, Spain
23	Immunotherapy With ex Vivo Expanded Haploidentical Natural Killer Cells for Children/Young Adults With AML	Recruiting	No Results Available	•Acute Myeloid Leukemia	•Biological: NK cell infusions	•Belarussian Research Center for Pediatric Oncology, Hematology and Immunology, Minsk, Minsk Region, Belarus
24	NK Cell Therapy for AML	Recruiting	No Results Available	•AML	•Drug: CT101a	•The First Affiliated Hospital#College of Medicine, Zhejiang University, Hangzhou, Zhejiang, China
25	Ex Vivo Expanded NK Cells Infusion Decrease Relapse Post Hematopoietic Stem Cell Transplantation	Recruiting	No Results Available	•Hematologic Malignancy	•Procedure: Allogeneic Hematopoietic Stem Cell Transplantation	•West China Hospital of Sichuan University, Chengdu, Sichuan, China
26	NKG2D CAR-T Cells to Treat Patients With Previously Treated Liver Metastatic Colorectal Cancer	Recruiting	No Results Available	•Refractory Metastatic Colorectal Cancer	•Biological: CAR-T infusion	•The Third Affiliated Hospital of Guangzhou Medical University, Guangzhou, Guangdong, China
27	NKG2D CAR-NK Cell Therapy in Patients With Relapsed or Refractory Acute Myeloid Leukemia	Recruiting	No Results Available	•Safety and Efficacy	•Biological: CAR-NK cells	•Hebei Yanda Lu Daopei Hospital, Sanhe, Hebei, China
28	Study of Anti-CD33/CLL1 CAR-NK in Acute Myeloid Leukemia	Recruiting	No Results Available	•Acute Myeloid Leukemia	•Biological: Anti-CD33/CLL1 CAR-NK Cells	•Wuxi People's Hospital, Wuxi, Jiangsu, China
29	NKG2D CAR-NK Cell Therapy in Patients With Refractory Metastatic Colorectal Cancer	Recruiting	No Results Available	•Refractory Metastatic Colorectal Cancer	•Drug: NKG2D CAR-NK	•The First Affiliated Hospital, Zhejiang University, Hangzhou, Zhejiang, China
30	Study of Anti-5T4 CAR-NK Cell Therapy in Advanced Solid Tumors	Recruiting	No Results Available	•Advanced Solid Tumors	•Biological: Anti-CAR-NK Cells	•Wuxi People's Hospital, Wuxi, Jiangsu, China
31	Camrelizumab in Combination With Apatinib Plus NK Cell for Advanced HCC	Recruiting	No Results Available	•Hepatocellular Carcinoma	•Drug: Apatinib<br>•Drug: Camrelizumab<br>•Drug: NK cell	•Nanfang Hospital of Southern Medical University, Guangzhou, Guangdong, China
32	Treatment of Malignant Tumors With NK Cell	Recruiting	No Results Available	•Malignancy	•Biological: Decitabine combined with NK cell infusion	•Shenzhen University General hospital, Shenzhen, Guangdong, China
33	Nk Cell Therapy for Recurrent Glioblastoma Multiform Patients	Recruiting	No Results Available	•Glioblastoma Multiform<br>•Recurrent Glioblastoma	•Biological: NK cell therapy	•Royan institute, Tehran, Iran, Islamic Republic of
34	Phase I/II Study of CAR.70- Engineered IL15-transduced Cord Blood-derived NK Cells in Conjunction With Lymphodepleting Chemotherapy for the Management of Relapse/Refractory Hematological Malignances	Recruiting	No Results Available	•B-Cell Lymphoma<br>•Myelodysplastic Syndromes (MDS)<br>•Acute Myeloid Leukemia (AML)	•Drug: Cyclophosphamide<br>•Drug: CAR.70/IL15-transduced CB-NK cells<br>•Drug: Fludarabine phosphate	•M D Anderson Cancer Center, Houston, Texas, United States
35	Natural Killer (NK) Cell Therapy in Locally Advanced HCC	Recruiting	No Results Available	•Locally Advanced Hepatocellular Carcinoma	•Biological: Vax-NK/HCC	•Seon-Ah Ha, Hwasun, Jeollanam-do, Korea, Republic of
36	Anti-CD33 CAR NK Cells in the Treatment of Relapsed/ Refractory Acute Myeloid Leukemia	Recruiting	No Results Available	•Leukemia, Myeloid, Acute	•Biological: anti-CD33 CAR NK cells<br>•Drug: Fludarabine<br>•Drug: Cytoxan	•Department of Hematology, Xinqiao Hospital, Chongqing, Chongqing, China

	Title	Status	Study Results	Conditions	Interventions	Locations
37	Anti-BCMA CAR-NK Cell Therapy for the Relapsed or Refractory Multiple Myeloma	Recruiting	No Results Available	•Multiple Myeloma, Refractory	•Biological: Anti-BCMA CAR-NK Cells<br>•Drug: Fludarabine<br>•Drug: Cytoxan	•Department of Hematology, Xinqiao Hospital, Chongqing, Chongqing, China
38	Safety and Efficacy of Allogeneic NK Cell Infusions in Patients With Relapsed/Refractory AML and High Risk MDS	Recruiting	No Results Available	•AML, Adult Recurrent<br>•MDS	•Biological: DVX201	•Duke University Hospital, Durham, North Carolina, United States
39	Clinical Study of HLA Haploidentical CAR-NK Cells Targeting CD19 in the Treatment of Refractory/Relapsed B-cell NHL	Recruiting	No Results Available	•B-cell Non Hodgkin Lymphoma	•Biological: anti-CD19 CAR-NK	•2nd Affiliated Hospital, School of Medicine, Zhejiang University, Hangzhou, Zhejiang, China
40	A Study to Evaluate the Safety and Anti-tumor Activity of SNK01 (NK Cells) Administered in Combination With Chemotherapy or Chemotherapy / Cetuximab in Local Advanced or Metastatic Non-small Cell Lung Cancer Patients Who Failed Tyrosine Kinase Inhibitor Treatment	Recruiting	No Results Available	•Non-small Cell Lung Cancer	•Biological: SNK01 (Super Natural Killer Cells 01)<br>•Drug: GC<br>•Biological: Cetuximab	•Asan Medical Center, Seoul, Songpa-gu, Korea, Republic of
41	Third-Party Natural Killer Cells and Mogamulizumab for the Treatment of Relapsed or Refractory Cutaneous T-cell Lymphomas or Adult T-Cell Leukemia/Lymphoma	Recruiting	No Results Available	•Recurrent Adult T-Cell Leukemia/ Lymphoma<br>•Recurrent Primary Cutaneous T-Cell Non-Hodgkin Lymphoma<br>•Refractory Adult T-Cell Leukemia/ Lymphoma<br>•Refractory Primary Cutaneous T-Cell Non-Hodgkin Lymphoma	•Drug: Cyclophosphamide<br>•Drug: Fludarabine<br>•Biological: Mogamulizumab<br>•Biological: Natural Killer Cell Therapy<br>•Other: Quality-of-Life Assessment<br>•Other: Questionnaire Administration	•Ohio State University Comprehensive Cancer Center, Columbus, Ohio, United States
42	Immunotherapy Combination: Irradiated PD-L1 CAR-NK Cells Plus Pembrolizumab Plus N-803 for Subjects With Recurrent/ Metastatic Gastric or Head and Neck Cancer	Recruiting	No Results Available	•Gastroesophageal Junction (GEJ) Cancers<br>•Advanced HNSCC	•Drug: N-803<br>•Drug: Pembrolizumab<br>•Biological: PD-L1 t-haNK	•National Institutes of Health Clinical Center, Bethesda, Maryland, United States
43	Cord Blood Derived Anti-CD19 CAR-Engineered NK Cells for B Lymphoid Malignancies	Recruiting	No Results Available	•Acute Lymphocytic Leukemia<br>•Chronic Lymphocytic Leukemia<br>•Non Hodgkin's Lymphoma	•Drug: Fludarabine + Cyclophosphamide + CAR-NK-CD19 Cells	•Union Hospital, Huazhong University of Science and Technology, Wuhan, Hubei, China
44	Autologous Memory-like NK Cell Therapy With BHV-1100 (Formerly KP1237), Low Dose IL-2 in Multiple Myeloma Patients	Recruiting	No Results Available	•Multiple Myeloma	•Combination Product: CIML NK Cells plus KP1237 and low dose IL-2	•Dana Farber Cancer Institute, Boston, Massachusetts, United States
45	NKX101, Intravenous Allogeneic CAR NK Cells, in Adults With AML or MDS	Recruiting	No Results Available	•Relapsed/Refractory AML<br>•AML, Adult<br>•MDS<br>•Refractory Myelodysplastic Syndromes	•Biological: NKX101 - CAR NK cell therapy	•Colorado Blood Cancer Institute, Denver, Colorado, United States<br>•Winship Cancer Institute, Emory University, Atlanta, Georgia, United States<br>•University of Chicago Medical Center, Chicago, Illinois, United States<br>•The Cleveland Clinic - Taussig Cancer Institute, Cleveland, Ohio, United States<br>•Sarah Cannon at TriStar Bone Marrow Transplant Center, Nashville, Tennessee, United States<br>•MD Anderson Cancer Center, University of Texas, Houston, Texas, United States<br>•Methodist Healthcare System of San Antonio, San Antonio, Texas, United States
46	Allogeneic PB103 (NK Cells) Therapy in Non-small Cell Lung Cancer (NSCLC) Patients	Recruiting	No Results Available	•Non-small Cell Lung Cancer	•Biological: donor-derived NK cell infusion	•Tri-Service General Hospital, Taipei city, Taiwan
47	Clinical Trial for Autologus NK Cells Alone or in Combination With Isatuximab as Maintenance for Multiple Myeloma	Recruiting	No Results Available	•Multiple Myeloma	•Drug: CellProtect<br>•Drug: Isatuximab	•Karolinska University Hospital, Huddinge, Stockholm, Sweden

	Title	Status	Study Results	Conditions	Interventions	Locations
48	Clinical Study of Autologous Natural Killer Cells in Multiple Myeloma	Active, not recruiting	No Results Available	•Multiple Myeloma	•Drug: autologous NK cells	•Karolinska University Hospital, Stockholm, Sweden
49	Natural Killer-cell Therapy for Acute Myeloid Leukemia	Recruiting	No Results Available	•Acute Myeloid Leukemia Refractory<br>•Acute Myeloid Leukemia, Relapsed, Adult	•Biological: UCB-NK cells<br>•Drug: IL-2	•Radboud University Medical Center, Nijmegen, Netherlands
50	Natural Killer Cell (CYNK-001) Infusions in Adults With AML	Recruiting	No Results Available	•Leukemia<br>•Leukemia, Myeloid<br>•Leukemia, Myeloid, Acute<br>•Neoplasms by Histologic Type<br>•Neoplasms<br>•Immunosuppressive Agents<br>•Immunologic Factors<br>•Physiological Effects of Drugs<br>•Alkylating Agents<br>•Antimetabolites, Antineoplastic<br>•and 10 more	•Biological: CYNK-001	•Colorado Blood Cancer Institute, Denver, Colorado, United States<br>•University of Chicago, Chicago, Illinois, United States<br>•Hackensack University Medical Center, Hackensack, New Jersey, United States<br>•Roswell Park Comprehensive Cancer Center, Buffalo, New York, United States<br>•Columbia University and New York Presbyterian Hospital, New York, New York, United States<br>•Memorial Sloan Kettering Cancer Center, New York, New York, United States<br>•Westchester Medical Center, Valhalla, New York, United States<br>•Tennessee Oncology, Nashville, Tennessee, United States<br>•MD Anderson Cancer Center, Houston, Texas, United States<br>•Swedish Health Services, Seattle, Washington, United States
51	Natural Killer Cell (CYNK-001) Infusions in Adults With Multiple Myeloma	Active, not recruiting	No Results Available	•Multiple Myeloma<br>•Neoplasm, Plasma Cell<br>•Neoplasms by Histologic Type<br>•Neoplasms<br>•Hemostatic Disorder<br>•Vascular Diseases<br>•Cardiovascular Diseases<br>•Paraproteinemias<br>•Blood Protein Disorders<br>•Hematologic Diseases<br>•and 10 more	•Biological: CYNK-001	•Colorado Blood Cancer Institute, Denver, Colorado, United States<br>•Moffitt Cancer Center, Tampa, Florida, United States<br>•Franciscan Health, Indianapolis, Indiana, United States<br>•Washington University, Saint Louis, Missouri, United States<br>•University of Nebraska Medical Center, Omaha, Nebraska, United States<br>•Hackensack University Medical Center, Hackensack, New Jersey, United States<br>•Roswell Park Comprehensive Cancer Institute, Buffalo, New York, United States<br>•Oregen Health Knight Cancer Institute, Portland, Oregon, United States<br>•Tennessee Oncology, Nashville, Tennessee, United States
52	Ph1 Trial Test Safety of IL-21 NK Cells for Induction of R/R AML	Recruiting	No Results Available	•Allogeneic Stem Cell Transplant Recipient<br>•Blasts 10 Percent or More of Bone Marrow Nucleated Cells<br>•Recurrent Acute Myeloid Leukemia<br>•Refractory Acute Myeloid Leukemia	•Drug: Cytarabine Hydrochloride<br>•Drug: Fludarabine<br>•Biological: Membrane-bound Interleukin-21-Expanded Haploidentical Natural Killer Cells	•Ohio State University Comprehensive Cancer Center, Columbus, Ohio, United States
53	NK Cells Infusions With Irinotecan, Temozolomide, and Dinutuximab	Recruiting	No Results Available	•Relapsed Neuroblastoma<br>•Refractory Neuroblastoma	•Biological: Natural Killer Cells<br>•Drug: Temozolomide<br>•Drug: Irinotecan<br>•Drug: Dinutuximab<br>•Drug: Sargramostim	•Nationwide Children's Hospital, Columbus, Ohio, United States
54	Haploidentical Stem Cell Transplant With or Without NK Cell Infusion in AML and MDS	Recruiting	No Results Available	•Acute Myeloid Leukemia<br>•Myelodysplasia	•Biological: Haplo SCT with NK cells<br>•Biological: Haplo SCT	•Fondazione Policlinico Universitario A. Gemelli IRCCS, Roma, RM, Italy

	Title	Status	Study Results	Conditions	Interventions	Locations
55	Safety and Efficacy of Allogeneic NK Cells Therapy in Patients With Advanced Hepatocellular Carcinoma	Recruiting	No Results Available	•Hepatocellular Carcinoma	•Biological: allogeneic NK cells therapy	•The Fifth Medical Center of PLA General Hospital, Beijing, Beijing, China
56	A Phase 1 Trial of CIML NK Cell Infusion for Myeloid Disease Relapse After Hematopoietic Cell Transplantation	Recruiting	No Results Available	•Acute Myeloid Leukemia<br>•Myelodysplastic Syndromes<br>•Myeloproliferative Neoplasm<br>•Juvenile Myelomonocytic Leukemia	•Biological: CIML NK<br>•Drug: Fludarabine<br>•Drug: Cyclophosphamide	•Boston Children's Hospital, Boston, Massachusetts, United States<br>•Dana Farber Cancer Institute, Boston, Massachusetts, United States
57	Intraperitoneal Infusion of ex Vivo-cultured Allogeneic NK Cells in Recurrent Ovarian Carcinoma Patients	Recruiting	No Results Available	•Recurrent Ovarian Carcinoma<br>•Recurrent Fallopian Tube Carcinoma<br>•Recurrent Primary Peritoneal Carcinoma	•Biological: UCB-NK cells<br>•Drug: Chemotherapy	•Radboudumc, Nijmegen, Netherlands
58	Donor Natural Killer Cells, Cyclophosphamide, and Etoposide in Treating Children and Young Adults With Relapsed or Refractory Solid Tumors	Recruiting	No Results Available	•Recurrent Cutaneous Melanoma<br>•Recurrent Lip and Oral Cavity Carcinoma<br>•Recurrent Malignant Endocrine Neoplasm<br>•Recurrent Malignant Female Reproductive System Neoplasm<br>•Recurrent Malignant Male Reproductive System Neoplasm<br>•Recurrent Malignant Mesothelioma<br>•Recurrent Malignant Neoplasm of Multiple Primary Sites<br>•Recurrent Malignant Oral Neoplasm<br>•Recurrent Malignant Pharyngeal Neoplasm<br>•Recurrent Malignant Skin Neoplasm<br>•and 18 more	•Biological: Cord Blood-derived Expanded Allogeneic Natural Killer Cells<br>•Drug: Cyclophosphamide<br>•Drug: Etoposide	•M D Anderson Cancer Center, Houston, Texas, United States
59	Safety and Feasibility of the Use of Natural Killer Cells in Patients With Chronic Myeloid Leukemia	Enrolling by invitation	No Results Available	•Chronic Myeloid Leukemia	•Biological: Chronic Myeloid Leukemia + NK cell	•Centro Terapia e Tecnologia Celular, Porto Alegre, Rio Grande Do Sul, Brazil
60	Expanded Natural Killer Cells Following Haploidentical HSCT for AML/MDS	Recruiting	No Results Available	•Acute Myeloid Leukemia<br>•Myelodysplastic Syndromes	•Other: NK-DLI	•University Hospital Basel, Basel, Switzerland
61	Cytokine Induced Memory-like NK Cell Adoptive Therapy for Relapsed AML After Allogeneic Hematopoietic Cell Transplant	Recruiting	No Results Available	•Acute Myeloid Leukemia in Children	•Drug: CIML NK Cell Infusion<br>•Procedure: CD3+ T Cell Product Infusion	•Washington University School of Medicine, Saint Louis, Missouri, United States
62	Umbilical & Cord Blood (CB) Derived CAR-Engineered NK Cells for B Lymphoid Malignancies	Active, not recruiting	No Results Available	•B-Lymphoid Malignancies<br>•Acute Lymphocytic Leukemia<br>•Chronic Lymphocytic Leukemia<br>•Non-hodgkin Lymphoma	•Drug: Fludarabine<br>•Drug: Cyclophosphamide<br>•Drug: Mesna<br>•Biological: iC9/CAR.19/IL15-Transduced CB-NK Cells<br>•Drug: AP1903	•University of Texas MD Anderson Cancer Center, Houston, Texas, United States

	Title	Status	Study Results	Conditions	Interventions	Locations
63	Phase I Trial of Universal Donor NK Cell Therapy in Combination With ALT803	Active, not recruiting	No Results Available	•Acute Myeloid Leukemia<br>•Myelodysplastic Syndrome<br>•Acute Lymphoblastic Leukemia<br>•Chronic Myeloid Leukemia<br>•Chronic Lymphocytic Leukemia<br>•Non Hodgkin Lymphoma<br>•Hodgkin Lymphoma<br>•Myeloproliferative Syndromes<br>•Plasma Cell Myeloma<br>•Colon Carcinoma<br>•and 4 more	•Biological: Natural Killer (NK) Cells<br>•Biological: ALT803	•University Hospitals Cleveland Medical Center, Case Comprehensive Cancer Center, Cleveland, Ohio, United States
64	Cytokine Induced Memory-like NK Cell Adoptive Therapy After Haploidentical Donor Hematopoietic Cell Transplantation	Recruiting	No Results Available	•Acute Myeloid Leukemia	•Procedure: Graft cell infusion<br>•Drug: Tacrolimus<br>•Drug: Mycophenolate mofetil<br>•Drug: G-CSF<br>•Procedure: CIML NK cell infusion<br>•Drug: ALT-803<br>•Procedure: Leukapheresis	•Washington University School of Medicine, Saint Louis, Missouri, United States
65	Personalized NK Cell Therapy in CBT	Recruiting	No Results Available	•Accelerated Phase Chronic Myelogenous Leukemia, BCR-ABL1 Positive<br>•Acute Biphenotypic Leukemia<br>•Acute Lymphoblastic Leukemia<br>•Acute Lymphoblastic Leukemia in Remission<br>•Acute Myeloid Leukemia With Myelodysplasia-Related Changes<br>•Acute Myeloid Leukemia With Variant MLL Translocations<br>•B Acute Lymphoblastic Leukemia With t(9;22)(q34.1;q11.2); BCR-ABL1<br>•Chemotherapy-Related Leukemia<br>•Chronic Myelomonocytic Leukemia<br>•Chronic Phase Chronic Myelogenous Leukemia, BCR-ABL1 Positive<br>•and 16 more	•Biological: Allogeneic Natural Killer Cell Line NK-92<br>•Biological: Anti-Thymocyte Globulin<br>•Drug: Busulfan<br>•Drug: Clofarabine<br>•Drug: Cyclophosphamide<br>•Drug: Fludarabine Phosphate<br>•Other: Laboratory Biomarker Analysis<br>•Drug: Melphalan<br>•Biological: Rituximab<br>•Radiation: Total-Body Irradiation<br>•Procedure: Umbilical Cord Blood Transplantation	•M D Anderson Cancer Center, Houston, Texas, United States
66	Humanized Anti-GD2 Antibody Hu3F8 and Allogeneic Natural Killer Cells for High-Risk Neuroblastoma	Active, not recruiting	No Results Available	•Neuroblastoma<br>•High-Risk	•Drug: cyclophosphamide<br>•Biological: NK cells<br>•Biological: hu3F8<br>•Drug: rIL-2	•Memorial Sloan Kettering Cancer Center, New York, New York, United States

	Title	Status	Study Results	Conditions	Interventions	Locations
67	Immunotherapy of Relapsed Refractory Neuroblastoma With Expanded NK Cells	Active, not recruiting	No Results Available	•Neuroblastoma	•Drug: Dinutuximab<br>•Biological: NK Cells<br>•Drug: Lenalidomide	•Children's Hospital Los Angeles, Los Angeles, California, United States<br>•UCSF Helen Diller Family Comprehensive Cancer Center, San Francisco, California, United States<br>•AFLAC Cancer Center and Blood Disorders Service of Children's Healthcare of Atlanta - Egleston Campus, Atlanta, Georgia, United States<br>•University of Chicago Comer Children's Hospital, Chicago, Illinois, United States<br>•Childrens Hospital Boston, Dana-Farber Cancer Institute., Boston, Massachusetts, United States<br>•C.S Mott Children's Hospital, Ann Arbor, Michigan, United States<br>•Cincinnati Children's Hospital Medical Center, Cincinnati, Ohio, United States<br>•Nationwide Children's Hospital, Columbus, Ohio, United States<br>•Children's Hospital of Philadelphia, Philadelphia, Pennsylvania, United States<br>•Cook Children's Healthcare System, Fort Worth, Texas, United States<br>•Seattle Children's Hospital, Seattle, Washington, United States
68	Cord Blood Natural Killer (NK) Cells in Leukemia/Lymphoma	Active, not recruiting	No Results Available	•Leukemia	•Drug: Lenalidomide<br>•Drug: Rituximab<br>•Drug: Fludarabine<br>•Drug: Cyclophosphamide<br>•Procedure: NK Cells<br>•Drug: Cytarabine	•University of Texas MD Anderson Cancer Center, Houston, Texas, United States
69	Phase I Study of Adoptive Immunotherapy With Enriched and Expanded Autologous Natural Killer (NK) Cells for Patients With Ph+ Acute Lymphoblastic Leukemia (ALL)	Active, not recruiting	No Results Available	•Acute Lymphoblastic Leukemia<br>•Complete Hematologic Remission (CHR)<br>•Persistent/Recurrent Minimal Residual Disease (MRD)	•Other: Autologous NK cells infusions	•ISS/AIFA, Roma, Italy<br>•Ospedale S. Eugenio, Roma, Italy<br>•Università Cattolica del Sacro Cuore - Policlinico A. Gemelli, Roma, Italy<br>•Università degli Studi "Sapienza" - Dip Biotecnologie Cellulari ed Ematologia - Divisione di Ematologia, Roma, Italy<br>•Università degli Studi - Policlinico di Tor Vergata, Roma, Italy<br>•Università Degli Studi Di Roma "Sapienza" - Dipartimento Di Medicina Traslazionale E Di Precisione - U.O.C. Ematologia, Roma, Italy
70	Phase 2 STIR Trial: Haploidentical Transplant and Donor Natural Killer Cells for Solid Tumors	Active, not recruiting	No Results Available	•Ewing Sarcoma<br>•Neuroblastoma<br>•Rhabdomyosarcoma<br>•Osteosarcoma<br>•CNS Tumors	•Procedure: Allogeneic HCT<br>•Drug: Donor NK Cell Infusion	•Children's Hospital of Wisconsin, Milwaukee, Wisconsin, United States<br>•Froedtert and The Medical College of Wisconsin, Milwaukee, Wisconsin, United States

	Title	Status	Study Results	Conditions	Interventions	Locations
71	Cytokine-induced Memory-like NK Cells in Patients With Acute Myeloid Leukemia (AML) or Myelodysplastic Syndrome (MDS)	Active, not recruiting	No Results Available	•Leukemia, Myeloid, Acute	•Drug: Fludarabine<br>•Drug: Cyclophosphamide<br>•Procedure: Leukapheresis<br>•Biological: Cytokine-induced killer cells<br>•Biological: IL-2<br>•Drug: ALT-803<br>•Procedure: Peripheral blood for correlative studies<br>•Procedure: Bone marrow for correlative studies	•Washington University School of Medicine, Saint Louis, Missouri, United States

U.S. National Library of Medicine | U.S. National Institutes of Health | U.S. Department of Health & Human Services
